# Accuracy of Genomic Prediction for Milk Production Traits in Philippine Dairy Buffaloes

**DOI:** 10.3389/fgene.2021.682576

**Published:** 2021-10-28

**Authors:** Jesus Rommel V. Herrera, Ester B. Flores, Naomi Duijvesteijn, Nasir Moghaddar, Julius H. van der Werf

**Affiliations:** ^1^ School of Environmental and Rural Science, University of New England, Armidale, NSW, Australia; ^2^ Philippine Carabao Center- University of the Philippines Los Banos, Laguna, Philippines; ^3^ Philippine Carabao Center National Headquarters, Muñoz, Philippines

**Keywords:** dairy buffalo, ssGBLUP, bias, accuracy of genomic prediction, pBLUP, GBLUP

## Abstract

The objective of this study was to compare the accuracies of genomic prediction for milk yield, fat yield, and protein yield from Philippine dairy buffaloes using genomic best linear unbiased prediction (GBLUP) and single-step GBLUP (ssGBLUP) with the accuracies based on pedigree BLUP (pBLUP). To also assess the bias of the prediction, the regression coefficient (slope) of the adjusted phenotypes on the predicted breeding values (BVs) was also calculated. Two data sets were analyzed. The GENO data consisting of all female buffaloes that have both phenotypes and genotypes (*n* = 904 with 1,773,305-days lactation records) were analyzed using pBLUP and GBLUP. The ALL data, consisting of the GENO data plus females with phenotypes but not genotyped (*n* = 1,975 with 3,821,305-days lactation records), were analyzed using pBLUP and ssGBLUP. Animals were genotyped with the Affymetrix 90k buffalo genotyping array. After quality control, 60,827 single-nucleotide polymorphisms were used for downward analysis. A pedigree file containing 2,642 animals was used for pBLUP and ssGBLUP. Accuracy of prediction was calculated as the correlation between the predicted BVs of the test set and adjusted phenotypes, which were corrected for fixed effects, divided by the square root of the heritability of the trait, corrected for the number of lactations used in the test set. To assess the bias of the prediction, the regression coefficient (slope) of the adjusted phenotypes on the predicted BVs was also calculated. Results showed that genomic methods (GBLUP and ssGBLUP) provide more accurate predictions compared to pBLUP. Average GBLUP and ssGBLUP accuracies were 0.24 and 0.29, respectively, whereas average pBLUP accuracies (for GENO and ALL data) were 0.21 and 0.22, respectively. Slopes of the two genomic methods were also closer to one, indicating lesser bias, compared to pBLUP. Average GBLUP and ssGBLUP slopes were 0.89 and 0.84, respectively, whereas the average pBLUP (for GENO and ALL data) slopes were 0.80 and 0.54, respectively.

## Introduction

The Philippine Carabao Center (PCC) has put in place a genetic improvement program that includes a system of evaluating genetically superior individual animals for milk and milk component traits and maintenance of nucleus herds of dairy buffaloes as source of breeding animals and provision of frozen semen from the best riverine buffalo germplasm (identified through progeny testing) for artificial insemination (AI). PCC maintains 12 institutional herds of dairy buffaloes [mostly Bulgarian Murrahs (BUL)] dispersed throughout the archipelago as source of breeding animals and frozen semen from the best riverine buffalo germplasm for AI to riverine, crossbred, and swamp buffaloes. Recording and evaluation of performance are presently limited to animals in these herds, numbering ∼1,200 females, of which ∼400 can be considered as elite dams (open-nucleus scheme). However, present constraints of the breeding program are as follows: the number of recorded cows is not expected to increase substantially in the immediate future; currently progeny is testing only eight bulls per year; accuracies of progeny test bulls are low due to small number of daughters with lactation records; and generation interval is long for AI sires, ∼8 years (Flores, 2014).

The availability of the Affymetrix 90K Buffalo Genotyping Array (Affymetrix, Inc., Santa Clara, CA) in 2013 made it possible to do genomic studies in the bubaline species (Iamartino et al., 2017). When the trait of interest cannot be recorded on the selection candidate, genomic selection schemes are very attractive even when the number of phenotypic records is limited, because traditional breeding requires progeny testing schemes with long generation intervals (Schaeffer, 2006). Having similarities with dairy cattle breeding, for example, long generation interval, traits that are sex-limited, and measured late in life, it is probable that the advantages of genomic selection seen in dairy cattle will also be observed in dairy buffalo.

Genomic prediction studies in dairy buffaloes are very limited and were based on small data sets. Tonhati et al. (2016) used single-step genomic best linear unbiased prediction (ssGBLUP) to estimate the predicted transmitting ability accuracies for seven milk traits on 452 Brazilian buffaloes. Using a fivefold cross-validation, Liu et al. (2017) evaluated the reliability of genomic estimated BVs and their correlation with EBVs for six milk production traits from 412 Italian Mediterranean (ITA) buffaloes.

The objective of this study was to determine the accuracy of genomic prediction and bias for milk yield (MY), fat yield (FY), and protein yield (PY) from Philippine dairy buffaloes using GBLUP and ssGBLUP compared to prediction accuracy and bias based on pedigree BLUP (pBLUP).

## Materials and Methods

Phenotype data and blood samples used in this study were obtained from the PCC. All animals are housed in institutional farms and cooperatives managed by PCC. Data collection and storage are managed by the center’s Animal Breeding and Genomics Section (ABGS).

### Phenotype Data

Traits investigated in this study are 305-days MY, FY, and PY. Descriptive statistics of the phenotypic data are presented in Tables 1 and 2. The numbers of animals with one, two, and three lactation records are shown in Tables 3 and 4.

**TABLE 1:** Descriptive statistics of GENO data to be used for pBLUP and GBLUP analyses.

Trait	No. of animals	No. of records	No. genotyped	Mean (kg)	Min (kg)	Max (kg)	SD (kg)
MY	904	1,773	904	1,573.2	103.1	3,054.5	505.9
FY	856	1,384	856	119.0	30.2	206.9	27.7
PY	856	1,384	856	70.7	22.5	127.9	16.0

MY, milk yield; FY, fat yield; PY, protein yield.

**TABLE 2:** Descriptive statistics of ALL data to be used for pBLUP and ssGBLUP analyses.

Trait	No. of animals	No. of records	No. genotyped	Mean (kg)	Min (kg)	Max (kg)	SD (kg)
MY	1,975	3,821	904	1,466.3	103.1	3,150.9	518.0
FY	1,918	3,405	856	111.9	29.3	210.1	29.1
PY	1,918	3,405	856	66.3	19.9	128.8	17.3

MY, milk yield; FY, fat yield; PY, protein yield.

**TABLE 3:** Number of animals (number of records) for test and training sets for MY.

Test set	Training set	
	GENO	ALL
329^a^ (329)	575 (1,444)	1,646 (3,492)
281^b^ (562)	623 (1,211)	1,694 (3,259)
294^c^ (882)	610 (891)	1,681 (2,939)

^a,b,c^Number of animals with 1, 2, and 3 lactation records, respectively.

**TABLE 4:** Number of animals (number of records) for test and training sets for FY and PY.

Test set	Training set	
	GENO	ALL
441^a^ (441)	415 (943)	1,477 (2,964)
302^b^ (604)	554 (780)	1,616 (2,801)
113^c^ (339)	743 (1,045)	1,805 (3,066)

^a,b,c^Number of animals with 1, 2, and 3 lactation records, respectively.

Two data sets were analyzed. One contains only female buffaloes that have both phenotypes and genotypes (hereby referred to as GENO) (Table 1). Analyses done on these data were pBLUP and GBLUP. The other data set (hereby referred to as ALL) (Table 2) contains all the above animals, plus females with phenotypes but are not genotyped. Analyses done on these data were pBLUP and ssGBLUP. A pedigree file containing 2,642 animals spanning six generations was used for pBLUP and ssGBLUP.

### Genotype Data

Genomic DNA was extracted using the Promega ReliaPrep Blood gDNA Miniprep System according to the manufacturer’s protocol. DNA quantification was done using the Promega Quantus Fluorometer. Samples were first subjected to RNA purification prior to shipment to Affymetrix, Inc. Submitted samples were genotyped using the Axiom 90k Buffalo Genotyping Array. Generated “.CEL” files were analyzed using the Axiom Analysis Suite using default settings, wherein polymorphic markers were identified. Additional quality control measures applied include a single-nucleotide polymorphism (SNP) removed if its minor allele frequency is less than 0.05, is out of Hardy-Weinberg equilibrium (*p* < 1 × 10^–15^), has no genome location, and is not found in the autosomes. After applying the quality control measures, only 60,827 SNPs in 29 autosomes were used for the determination of accuracy of genomic prediction and bias.

### Statistical Methods

BVs were estimated using three methods: pBLUP, GBLUP, and ssBLUP. The three methods used the following model:

$${305\mathrm{DTrait}}_{\mathrm{ijkp}}=\mu \;{+\;\mathrm{breed}}_{i}+\mathrm{lactation}\;{\mathrm{number}}_{j}{+\mathrm{HYS}}_{k}{\;+\;\mathrm{animal}}_{p}+\mathrm{permanent}\;{\mathrm{env}}_{p}{\;+\;e}_{\mathrm{ijkp}}$$

where *305dTrait* is a 305-days record for the desired trait (MY, FY, PY); *μ* is the general mean; *breed* is the fixed breed effect; *lactation number* is the fixed effect for lactation number; *HYS* is the fixed effect for herd-year-season; and *animal* and *permanent env* are the individual effect and permanent environmental effect on animal *p*; and *e* is random residual with *e* ∼ *N*(0,*e* ^2^).

The difference among the three methods is the type of relationships that was used. pBLUP uses a numerator relationship matrix (also known as an A-matrix) based on the pedigree (family relationships). The creation of the genomic relationship matrix (GRM), also known as the G-matrix, was used in GBLUP, and ssGBLUP is based on VanRaden (2008). The ssGLUP (Misztal et al., 2009; Legarra et al., 2014) uses an H-matrix (combination of family and genomic relationships), where the G-matrix replaces the A_22_ matrix (A-matrix containing only females that were genotyped).

### Validation Scheme

A threefold cross-validation scheme was used to compare accuracy of prediction and bias using GBLUP and ssGBLUP with those of pBLUP. Animals were assigned to one of three test sets: one lactation record, two lactation records, and three lactation records (Tables 3 and 4). One lactation record could mean that the animal has a record for the first lactation, second lactation, or third lactation. An animal with two lactation records could mean that it has the first two lactations, the first and the third lactations, or the second and third lactations. In each case, the training set is composed of animals in the data set that are not part of the test set. Phenotypes of animals in the test sets were masked, and BVs were then estimated for each set either by pBLUP and GBLUP for the GENO data or pBLUP and ssGBLUP for ALL data using ASReml 4.1 (Gilmour et al., 2015).

### Accuracy of Genomic Prediction

Accuracy of prediction was calculated as the correlation between the predicted BVs of the test set and its corresponding adjusted phenotypes, which were corrected for fixed effects, divided by the square root of the heritability of the trait, corrected for the number of lactations used in the test set:

$$r=\frac{\text{corr}\left(\text{BV},\text{adj}.\text{pheno}\right)}{\sqrt{\frac{h^{2}}{\text{rep}+\left(1-\;\frac{\text{rep}}{n}\right)\;}}}$$

where *r* is the accuracy of prediction; corr is the correlation; BV is the predicted BV; adj. pheno is the adjusted phenotype corrected for fixed effects; *h* ^2^ is the heritability of the trait; rep is the repeatability of test set; and *n* is the number of lactations records used in test set. Note that if *n* = 1, denominator is equal to *h*.

The average of the accuracies of the three test sets is the accuracy of prediction of a trait.

### Prediction Bias

To assess the bias of prediction, the regression coefficient (slope) of the adjusted phenotypes on the predicted BVs was also calculated, with slopes of approximately 1 showing zero bias. Slopes greater than or less than 1 indicate underestimation and overestimation, respectively, of BVs. The average of the slopes of the three test sets is the slope of a trait.

## Results

### Accuracy of Genomic Prediction

Accuracies of genomic prediction of the three traits through cross-validation are shown in Table 5. Heritabilities used are 0.19, 0.17, and 0.19 for MY, FY and PY, respectively, which were derived using pBLUP. Results showed that genomic methods (GBLUP and ssGBLUP) provide more accurate predictions compared to pBLUP. For the GENO data, GLUP accuracies increased for MY and FY by 0.08 and 0.01, respectively, whereas there was no increase for PY if compared to pBLUP accuracies. In the case of ALL data, ssGBLUP accuracies are higher by 0.13, 0.04, and 0.07 for MY, FY, and PY, respectively, if compared to pBLUP accuracies. Average pBLUP (for GENO and ALL data) accuracies for the three traits were 0.21 and 0.22, respectively, whereas the average GBLUP and ssGBLUP (for GENO and ALL data) accuracies were 0.24 and 0.29, respectively. GBLUP and ssGBLUP accuracies were, on average, 0.03 and 0.07 higher, respectively, compared to pBLUP accuracies.

**TABLE 5:** Accuracy of prediction for pBLUP, GBLUP, and ssGBLUP estimated from threefold cross-validation scheme.

Trait	GENO			ALL		
	pBLUP	GBLUP	Increase in accuracy	pBLUP	ssGBLUP	Increase in accuracy
MY	0.20 ± 0.04	0.28 ± 0.06	0.08	0.17 ± 0.02	0.30 ± 0.04	0.13
FY	0.23 ± 0.04	0.24 ± 0.05	0.01	0.26 ± 0.14	0.30 ± 0.01	0.04
PY	0.20 ± 0.05	0.20 ± 0.05	0	0.23 ± 0.14	0.26 ± 0.02	0.03
Average	0.21	0.24	0.03	0.22	0.29	0.07

MY, milk yield; FY, fat yield; PY, protein yield.

### Prediction Bias

In the case of bias of prediction, slopes for all methods were less than 1, indicating overestimation of BVs (Table 6). However, slopes of the two genomic methods are closer to 1, indicating lesser bias, compared to pBLUP slopes. Average pBLUP (for GENO and ALL data) slopes for the three traits were 0.80 and 0.54, respectively, whereas GBLUP and ssGBLUP slopes were 0.89 and 0.84, respectively.

**TABLE 6:** Estimated slopes calculated from breeding values from pBLUP, GBLUP, and ssGBLUP.

Trait	GENO		ALL	
	pBLUP	GBLUP	pBLUP	ssGBLUP
MY	0.69 ± 0.39	0.85 ± 0.28	0.42 ± 0.07	0.85 ± 0.16
FY	0.94 ± 0.17	0.99 ± 0.22	0.62 ± 0.36	0.88 ± 0.04
PY	0.76 ± 0.11	0.83 ± 0.34	0.57 ± 0.38	0.79 ± 0.10
Average	0.80	0.89	0.54	0.84

MY, milk yield; FY, fat yield; PY, protein yield.

## Discussion

With a limited number of progeny-tested bulls, a reference population of females with at most three lactations per animal was used in this study to determine the accuracy of genomic prediction and bias for MY, FY, and PY using GBLUP and ssGBLUP and compared to prediction accuracy and bias based on pedigree pBLUP. The accuracy of prediction was based on threefold cross-validation scheme (test sets are the number of lactations per animal), and bias was calculated as the regression coefficient (slope) of the adjusted phenotypes on the predicted BVs.

Several genomic prediction studies in dairy cattle have been done wherein the reference populations are cows. Brown et al. (2016) used crossbred cows from Kenya as no bulls were available that can be ranked because there is very little phenotypic and pedigree data available. In the case of Nayee et al. (2018), Holstein crossbred cows in India were used as the reference population because the annual numbers of progeny tested bulls are limited to 20 to 40 per year. With limited number of progeny-tested bulls with highly reliable EBV (reliability >0.8), Ding et al. (2013) established a reference population of Chinese Holstein females. In the case of dairy buffalo, two genomic prediction studies (Tonhati et al., 2016; Liu et al., 2017) were done based on small data sets of genotyped female buffaloes as the reference population.

**FIGURE 1:**
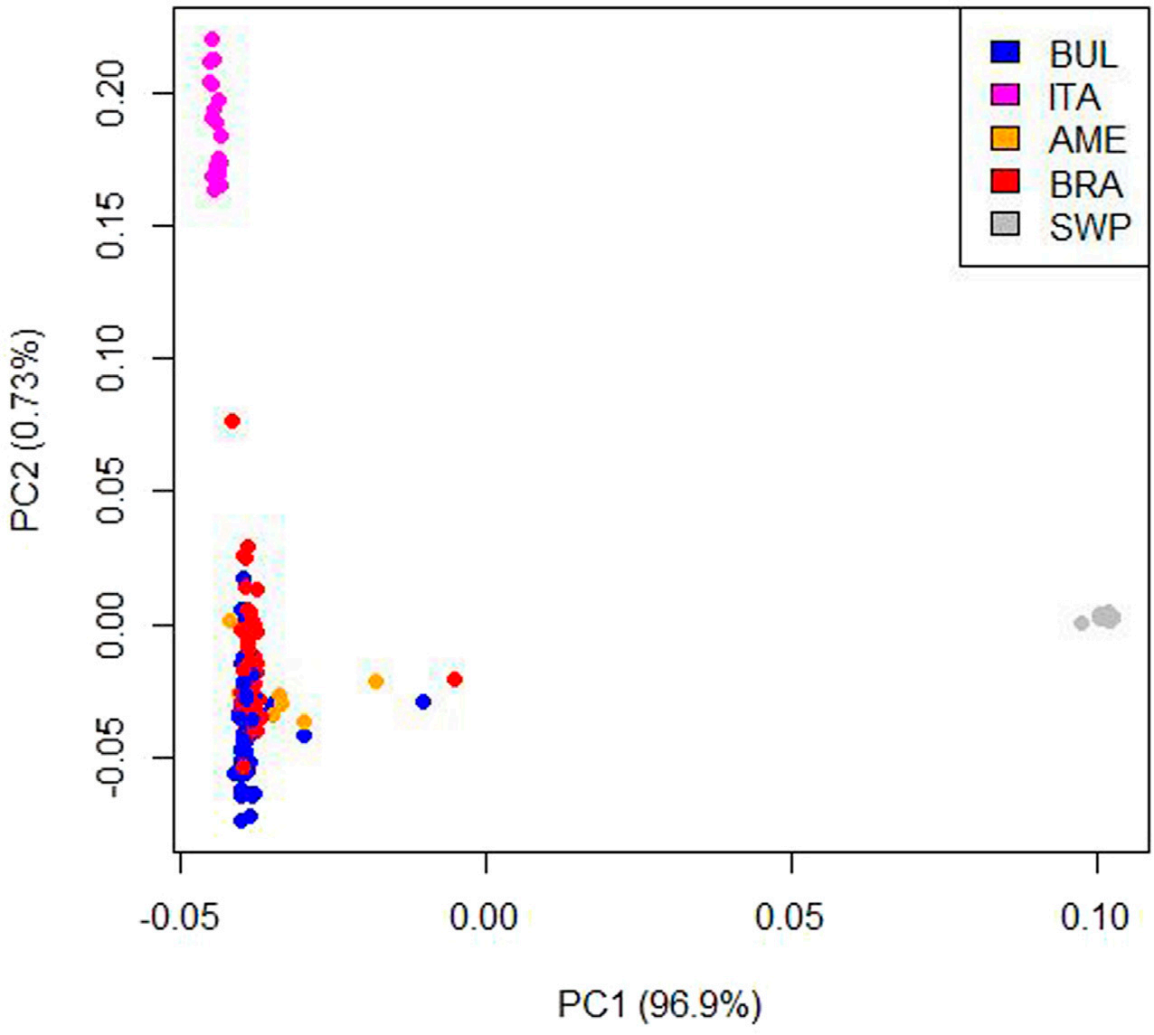
PCA plot generated based on the genomic relationship matrix of the five buffalo populations (*n* = 250). BUL, Bulgarian Murrah; BRA, Brazilian Murrah; ITA, Italian Mediterranean; AME, American Murrah; SWP, Philippine swamp.

Combining different breeds is another option to increase the reference population (Hayes et al., 2009; Cole and Silva, 2016). In this study, three breeds were included BUL, Brazilian Murrah (BRA), and American Murrah (AME). Based on their breed histories, these three breeds all have the riverine buffalo blood from India as ancestors. The BUL was created by crossing the Indian Murrah imported into Bulgaria in 1962 and 1975 with the native Bulgarian Mediterranean buffaloes (Alexiev, 1998; Borghese, 2013). Buffaloes imported by PCC from Brazil in 2013 were all Indian Murrah and their crosses. The AME came from one buffalo herd from Florida; the most probable source of the foundation stock came from the University of Florida, wherein in 1979, 14 cows and 2 bulls of the Bufalypso breed from Trinidad were delivered, which were created during 1949–1960 from 7 imported Indian buffalo breeds [(Alexiev, 1998). A principal component analysis (PCA) (Figure 1] was done in a previous study wherein these three breeds were grouped together. PCC also has an ITA buffalo population but was not included in this study as it formed a separate group in the PCA plot (Figure 1). Included also in the reference population are crosses of BUL bulls with BRA (BUL × BRA) and AME (BUL × BRA) females. Moreover, all the institutional herds, dispersed throughout the archipelago, are linked using BUL sires.

The increase in accuracy in GBLUP could be due to the realized relationships of animals in GBLUP compared to just expected relationships of animals in pBLUP. For example, full sibs would have an expected relationship of 0.5 in pBLUP, but this could be 0.3 to 0.6 in GBLUP. The increase in accuracy in ssGLUP could also be due to the above plus the linking of unrelated families, which is not possible with pBLUP. As an example, two families in pBLUP are not related because they do not share a common ancestor. In ssGBLUP, genotyping only one animal in each family would serve as a link between these two families; this relationship between these two genotyped animals will now create relationships among all animals in both families.

The accuracy of prediction for MY in this study using GBLUP and ssGBLUP was 0.28 and 0.30, respectively. These were lower than reported studies using dairy cows as the reference population. Brown et al. (2016) had an accuracy of prediction of 0.32–041 for MY using GBLUP with a reference population of 1,013 crossbred Kenyan cows. The creation of the GRM (G-matrix) here made it possible to estimate the genetic relationships among the animals, all of which do not have pedigree information. The accuracy of prediction of (Nayee et al., 2018) using ssGBLUP for MY was 0.387–0.405 with a larger reference population of 10,797 Holstein crossbred cows. In the case of Ding et al. (2018), accuracies of prediction for MY, FY, and PY were 0.37, 0.32, and 0.40, respectively, using 3,087 Chinese Holstein cows. In the case of dairy buffaloes, accuracies of prediction in Liu et al. (2017) are similar for MY (0.28), but higher for FY (0.35 vs. 0.24) and PY (0.24 vs. 0.20). The study by Liu et al. reported reliabilities, whereas accuracy is the square root of reliability.

A limitation of this study is the small data set. Female animals with production and genotype data will be added yearly to increase the reference population. Potential semen donor bulls will be genotyped to determine their BVs using the population of cows as the reference population.

### Implications

**FIGURE 2:**
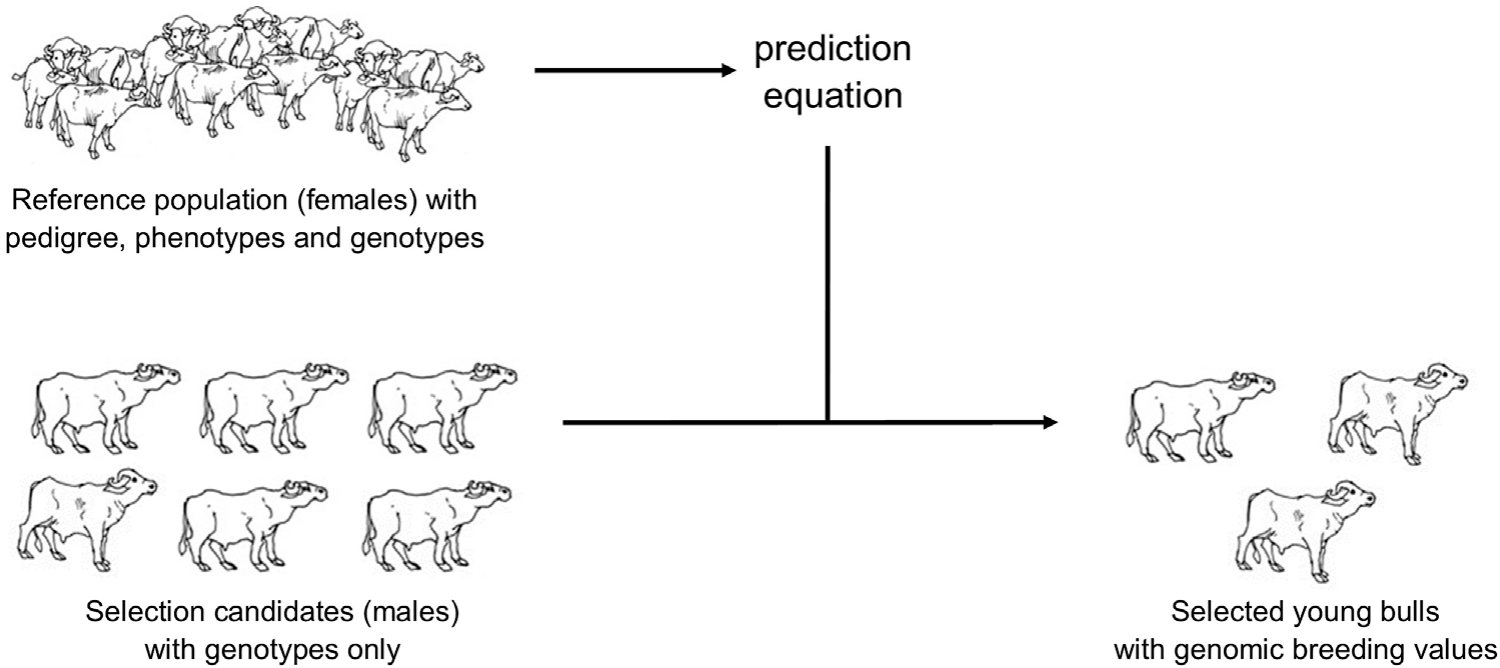
Genomic selection in Philippine dairy buffaloes.

At present, the generation interval of AI buffalo sires is ∼8 years. With GS, young genotyped candidate bulls can be given BVs using females in the institutional herds as the reference population (Figure 2). ssGBLUP method can be used to generate BVs as some females with performance data cannot be genotyped anymore (ie, dead). Moreover, limited funds allocated per year may not allow genotyping of all cows with at least one lactation record. Selected candidate bulls coming from the institutional herds (and cooperatives) that will be genotyped are closely related to the reference population as their female relatives (dams, granddams, siblings) are in that population. Young bulls can now be selected at a younger age; generation interval can be lowered to ∼3.5 years old. A future study will be done to compare the present progeny testing breeding scheme and a genomic breeding scheme, that is, GBLUP in terms of genetic gain and cost savings from the point of view of PCC as the breeding entity.

## Conclusions

This study determined the accuracy of genomic prediction and bias for MY, FY, and PY in Philippine dairy buffaloes wherein the reference population is composed solely of cows. GBLUP and ssGBLUP accuracies were, on average, 0.03 and 0.07 higher, respectively, compared to pBLUP accuracies. Moreover, prediction bias of the two genomic methods is lesser (closer to 1) compared to pBLUP. With the higher accuracy of prediction and lesser bias, it is suggested that PCC adopts the genomic method, that is, GLUP or ssGBLUP, in its genetic evaluation.

## Data Availability

The data analyzed in this study is subject to the following licenses/restrictions: The datasets for this article are not publicly available because these are the exclusive property of the Philippine Carabao Center. Requests to access these datasets should be directed to Ronnie D. Domingo, OIC-Executive Director, pcc-oed@mozcom.com.
